# Plant-Based Yogurt Using Rice Bran and Grain of Green Rice: Increasing the Protein Content of Raw Materials by Enzymatic Protein Extraction

**DOI:** 10.3390/foods15071169

**Published:** 2026-03-31

**Authors:** Tarathep Siripan, Hua Li, Sirithon Siriamornpun

**Affiliations:** 1 Department of Food Technology and Nutrition, Faculty of Technology, Mahasarakham University, Kantarawichai 44150, Maha Sarakham, Thailand; 67010853501@msu.ac.th; 2 Department of Cuisine and Nutrition, Yangzhou University, Yangzhou 225127, China; lihua216@yzu.edu.cn; 3 Key Laboratory of Chinese Cuisine Intangible Cultural Heritage Technology Inheritance, Ministry of Culture and Tourism, Yangzhou 225127, China; 4 Research Unit of Thai Food Innovation (TFI), Mahasarakham University, Kantarawichai 44150, Maha Sarakham, Thailand

**Keywords:** enzymatic extraction, functional properties, rheological properties, sensory evaluation, alternative yogurt

## Abstract

This study investigated rice bran and green Khao Dawk Mali 105 (KDML 105) rice grains as alternative protein sources for plant-based yogurt. However, there is a lack of systematic investigation on the application of enzyme-extracted proteins from green KDML 105 rice and rice bran in fermented yogurt systems. Proteins were obtained via enzyme-assisted extraction to enhance yield and bioactive compound release prior to formulation. Physicochemical, compositional, rheological, bioactive, and sensory properties were evaluated. Yogurts by green rice protein extract (GRE) and green rice bran protein extract (GBE) formed softer gel networks than soy yogurt, exhibiting lower water-holding capacity and higher syneresis, reflecting differences in protein aggregation during fermentation. Nevertheless, green rice (GR) and green rice bran (GB) yogurts contained significantly higher protein levels (1.93–2.47-fold) than the control. They also demonstrated enhanced bioactive properties, with increased total phenolic content (1.07–1.51-fold), total flavonoid content (2.10–4.35-fold), DPPH radical scavenging activity (1.07–1.16-fold), and FRAP values (1.46–1.98-fold). Sensory evaluation indicated good acceptability, particularly for GR formulations, which achieved a mean score of 7 with favorable texture and flavor attributes. These findings highlight the technological feasibility of utilizing green rice and rice bran proteins as primary ingredients in rice-based fermented yogurt alternatives with improved bioactive functionality.

## 1. Introduction

Yogurt is traditionally manufactured through the fermentation of milk, but in recent years it has increasingly been formulated using plant-based substrates, including almond, coconut, and oat beverages. This diversification reflects growing consumer interest in vegan and lactose-free alternatives, alongside broader dietary and sustainability considerations [1]. In addition to nutritional aspects, sensory properties, with emphasis on texture and flavor, contribute significantly to consumer acceptance of yogurt products. Yogurt fermentation is typically driven by a symbiotic association between *Streptococcus thermophilus* and *Lactobacillus delbrueckii* subsp. *bulgaricus*, which together are responsible for acid production and gel formation. Depending on the desired functional and sensory profile, additional microorganisms may be added [2,3].

Rice (*Oryza sativa* L.) is a major staple food for more than half of the global population, particularly in Asian regions [4,5]. Khao Dawk Mali 105 (KDML 105), despite its high market value, KDML 105 is predominantly commercialized as milled rice, with limited development into value-added or processed products [6]. Moreover, research addressing advanced or alternative applications of this cultivar remains relatively scarce, particularly for green Khao Dawk Mali 105 grains. Given its distinctive quality attributes and strong global demand, KDML 105 represents a relevant model for exploring rice quality and functional properties beyond conventional uses [7]. Green rice grains differ from fully mature grains in protein composition and functional behavior, which may influence their suitability for food processing applications [7,8]. In particular, green rice has been reported to contain higher levels of protein, bioactive compounds (such as phenols), and certain nutrients than cooked rice, which may result in improved nutritional value and functional properties of the final product [8,9,10].

Despite increasing interest in plant-based fermented foods, systematic investigations into the use of green KDML 105 rice protein in yogurt-type products remain limited. Most plant-based yogurt alternatives are still formulated primarily from legumes or nuts, while rice protein has received comparatively little attention as a fermentation substrate. In particular, studies on yogurt like products derived from aromatic rice varieties, such as Khao Dawk Mali 105 [11], are scarce. Furthermore, the application of green KDML 105 rice grains in fermented food systems has not been extensively explored, especially with respect to protein functionality, fermentation behavior, and their effects on final product quality.

Therefore, this study evaluates the potential of green Khao Dawk Mali 105 rice protein as a raw material for plant-based yogurt production. The effects of fermentation on physicochemical properties, functional characteristics, and overall product quality were investigated to assess its feasibility as a value-added, dairy-free yogurt alternative.

## 2. Materials and Methods

### 2.1. Materials and Chemicals

Green Khao Dawk Mali 105 (KDML 105) rice grains and corresponding green rice bran were harvested at approximately 22–28 days after pollination. The samples were obtained from Roi Et Province, Thailand, and stored at 4 °C until further analysis. The commercial plant-based yogurt (Soygurt, Original flavor) was used. Soybeans were purchased from a local retail market in Kantarawichai District, Maha Sarakham Province, Thailand. A food-grade protease preparation (80,000 IU/mL) was supplied by KANEZYME (Bangkok, Thailand). Food-grade sodium hydroxide was obtained from Krungthepchemi (Bangkok, Thailand). All reagents were of analytical grade and sourced from Sigma-Aldrich (St. Louis, MO, USA).

### 2.2. Preparation of Rice Protein Extracts for Plant-Based Yogurt Production

#### 2.2.1. Sample Preparation of Yogurt Production

Green rice grains and green rice bran were defatted following the method described by Wattanasiritham et al. [12], with minor modifications. To inactivate peroxidase activity and minimize lipid oxidation, rice bran samples were gently roasted at 70 °C prior to defatting [13]. The ground material was passed through a 60-mesh sieve (250 μm) and mixed with hexane at a ratio of 1:3 (*w*/*v*). Before use, hexane was completely removed from the samples using a rotary evaporator. The mixture was shaken for 1 h at room temperature and subsequently air-dried under a fume hood using a rotary evaporator to remove residual solvent. The defatted samples were then finely ground, subsequently sieved, and stored at 4 °C in aluminum foil pouches containing desiccant prior to further use.

#### 2.2.2. Enzymatic Approach to Protein Extraction

Protein extraction was performed according to the method of Siripan et al. [8], with slight modifications. Defatted samples (150 g) were suspended in 600 mL of distilled water, and the pH was adjusted to 6.5. The suspension was incubated at 50 °C, after which a food-grade protease was added at a concentration of 0.4% (*v*/*v*). Enzymatic hydrolysis was allowed to proceed under controlled conditions and was terminated by heating the mixture at 95 °C for 10 min. The extract was then cooled to 25 °C in a water bath and pasteurized at 73 °C. Insoluble residues and residual starch were removed by filtration through a sterile white cloth. The resulting protein extracts were collected in sterile containers and stored at 4 °C until further analysis. Protein extraction from rice bran followed the same procedure as that used for rice grains.

#### 2.2.3. Verification of Gram-Positive Microorganisms by Gram Staining

To obtain a culture of Gram-positive bacteria, a commercial plant-based yogurt (Soygurt, original flavor) was serially diluted in sterile normal saline and streaked onto MRS agar plates. The plates were incubated at 37 °C for 48 h under anaerobic conditions [14]. To ensure culture purity, morphologically distinct single colonies were carefully selected and repeatedly subcultured onto fresh MRS agar plates using the streak plate technique until uniform colonies were obtained. According to the product label, the starter culture contained *Lactobacillus delbrueckii* subsp. *Bulgaricus*, *Streptococcus thermophilus*, *Lactobacillus acidophilus*, *Lactobacillus acidophilus*, and *Bifidobacterium*. These isolates were subsequently verified as Gram-positive lactic acid bacteria prior to being used as the fermentation inoculum [15].

### 2.3. Production of Plant-Based Yogurt

**Figure 1:**
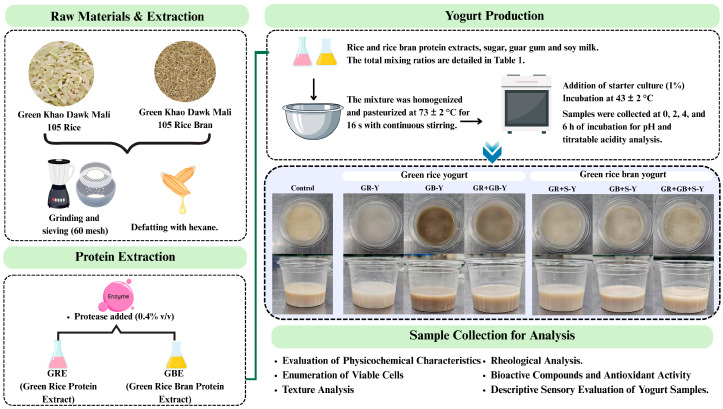
Protein extraction with protease enzyme and the plant-based yogurt production process.

Plant-based yogurts were prepared using a modified method from Hozzein et al. [16]. Seven formulations were developed: Control (soy milk, 92.90%), GR (green rice extract, 92.90%), GB (green rice bran extract, 92.90%), GR + GB (green rice extract 46.45% + green rice bran extract 46.45%), GR + S (green rice extract 46.45% + soy milk 46.45%), GB + S (green rice bran extract 46.45% + soy milk 46.45%), and GR + GB + S (soy milk 46.45% + green rice extract 23.225% + green rice bran extract 23.225%). All formulations were supplemented with 6% (*w*/*v*) sucrose, 0.1% (*w*/*v*) guar gum, and 1% (*v*/*v*) starter culture, resulting in an initial inoculum of approximately 2 × 10^7^ CFU/mL [17] (Table 1). The starter culture, indicated as *Lactobacillus delbrueckii* subsp. *Bulgaricus*, *Streptococcus thermophilus*, *Lactobacillus acidophilus*, *Lactobacillus acidophilus*, and *Bifidobacterium* was on the commercial product label. Mixtures were pasteurized at 73 ± 2 °C for 16 s with continuous stirring, cooled to 43 ± 2 °C, inoculated, and gently homogenized. Samples were incubated statically at 43 °C in sealed sterile containers (50 mL) to maintain a microaerophilic environment. The fermentation kinetics were monitored at 0, 2, 4, and 6 h by analyzing pH and titratable acidity. Fermentation was halted at 6 h by rapid cooling to 4 °C (when the pH reaches 4.5). Microbial growth was evaluated at the end of the fermentation period to ensure sufficient viable cell counts. A schematic diagram of the process is presented in Figure 1.

### 2.4. Evaluation of Physicochemical Characteristics

The physicochemical characteristics of the yogurt samples were evaluated by measuring pH, titratable acidity, and viable cell count using established analytical procedures.

#### 2.4.1. pH

The pH of the samples was measured using a calibrated pH meter after homogenization of 1 g of yogurt with 10 mL of distilled water. pH was measured using a benchtop pH meter (Mettler-Toledo F20-LE407, Greifensee, Switzerland) following the method of Mat Sarif et al. [18].

#### 2.4.2. Titratable Acidity

Titratable acidity was determined by titration with 0.1 M NaOH using phenolphthalein as an indicator (code 05173; Loba Chemie, Mumbai, India). Results were expressed as percentage of lactic acid and calculated according to Equation (1)

Total acidity (%) = mL × N × 90 × 100/V × 1000(1)

#### 2.4.3. Color Measurement

Color attributes (L*, a*, and b*) of the rice protein samples were measured using a Chroma Meter (CR-410, Konica Minolta Sensing Inc., Osaka, Japan), calibrated with a standard white ceramic tile prior to analysis.

#### 2.4.4. Measurement of Water-Holding Capacity (WHC)

Water-holding capacity (WHC) was evaluated following the method described by Boonarsa et al. [19], with minor modifications. Briefly, 0.5 g of sample was dispersed in 20 mL of distilled water and centrifuged at 4000 rpm for 10 min at 10 °C. The supernatant was discarded, the pellet was weighed, and WHC was calculated using Equation (2).

WHC (g/g) = W_initial (g)_ − W_final (g)_/W_initial (g)_ (2)

W_initial_ = Initial sample weight (g)

W_final_ = Final sample weight (g)

#### 2.4.5. Syneresis

Syneresis was determined following Lee and Lucey [20], with minor modifications. Yogurt samples (10 g) were transferred into centrifuge tubes and refrigerated at 4 °C for 24 h to allow full gel development. Samples were then centrifuged at 4000 rpm for 10 min at 4 °C. The expelled whey was carefully decanted and weighed, and syneresis was expressed as a percentage of the initial sample weight.

#### 2.4.6. Dry Matter, Ash, and Protein Analysis of Yogurt

Samples was performed in accordance with the Association of Official Analytical Chemists (AOAC) procedures (methods 925.23, 945.46, and 991.22) [19,20,21].

### 2.5. Enumeration of Viable Cells

The standard plate count method was applied to determine viable cell counts, using 1 mL of yogurt sample were serially diluted in 9 mL of sterile peptone water, and appropriate dilutions were spread onto MRS agar plates (Lactobacillus MRS Agar, GM641; HiMedia Laboratories, Mumbai, India). Incubation of the plates was carried out at 37 °C for a period of 48 h, after which colonies were counted. Viable cell counts were determined using MRS agar and expressed as total lactic acid bacteria (log CFU/mL). However, this method does not allow differentiation between individual LAB species and calculated according to Equation (3).

CFU/mL = Number of colonies counted/Volume of solution being counted (mL) × Dilution(3)

### 2.6. Texture Analysis

Texture analysis of yogurts was performed in triplicate using a TA-XT Plus texture analyzer (Stable Micro Systems, Godalming, UK), and firmness, consistency, cohesiveness, and viscosity index were determined through back extrusion with a 35-mm disc (A/BE rig, 1 kg load cell). Samples (≈75% filled) were tempered at 23 ± 2 °C for 30 min prior to analysis. Measurements were performed at a constant speed of 1.0 mm/s with a compression distance of 30 mm and a trigger force of 5 g [22].

### 2.7. Rheological Analysis

The rheological behavior of the samples was assessed using a controlled stress rheometer (HAAKE MARS 40, Thermo Fisher Scientific, Karlsruhe, Germany) with a 60-mm titanium parallel-plate configuration, (1-mm gap). Flow behavior was evaluated over a shear rate range of 0.1–100 s^−1^ at 25 ± 0.1 °C. Dynamic viscoelastic properties were obtained from frequency sweep tests (0.1–10 Hz), and storage modulus (G′), loss modulus (G″), and loss tangent (tan δ) were recorded [23].

#### Yield Stress Determination

Yield stress was determined under controlled stress conditions using a parallel plate geometry (PP60; 60 mm diameter, 1 mm gap). A logarithmic stress ramp (0.1–200 Pa) was applied for 120 s. The yield stress corresponded to the transition from elastic deformation to flow, as determined by the intersection of linear regions in the log–log plot of strain versus applied stress.

### 2.8. Bioactive Compounds and Antioxidant Activity

#### 2.8.1. Sample Preparation

Bioactive compounds were extracted following Siripan et al. [8], with minor modifications. Samples (1 g) were extracted with 20 mL of 80% (*v*/*v*) methanol at 37 °C for 20 h under agitation (150 rpm). Extracts were centrifuged (4000 rpm, 10 min), and supernatants were used for analysis. All samples were prepared on a dry weight basis to ensure analytical consistency and minimize variability associated with density-dependent measurements [24].

#### 2.8.2. Determination of Bioactive Compounds

The determination of total phenolic and flavonoid contents was carried out using the Folin–Ciocalteu method according to Boonarsa et al. [19]. Results were expressed as mg gallic acid equivalents for TPC and mg quercetin equivalents for TFC per 100 g dry weight.

#### 2.8.3. Determination of Antioxidant Activity

Antioxidant activity was evaluated using DPPH radical scavenging and ferric reducing antioxidant power (FRAP) assays, following Boonarsa et al. [19]. Results were expressed as mg L-ascorbic acid equivalents for DPPH and mg ferrous sulfate equivalents for FRAP per 100 g dry weight.

### 2.9. Descriptive Sensory Evaluation of Yogurt Samples

A descriptive sensory evaluation of yogurt samples was conducted by 30 untrained assessors (aged 18–45 years). Prior to participation, all assessors received information about the study and provided informed consent, (Human Research Ethics No. 429-046/2025), designed in accordance with international standards for sensory testing facilities [25]. A consensus-based approach, supervised by the panel leader, was applied to define the sensory attributes, their descriptions, and the evaluation procedure. The assessed attributes included appearance (color), smell, taste (sourness), texture, overall taste, and overall liking. Yogurt samples (20 mL) were served in clear plastic cups labeled with random three-digit codes and presented on white trays. Samples were equilibrated at room temperature (23 ± 2 °C) for 30 min prior to evaluation and assessed individually in sensory booths. All attributes were rated using a 9-point hedonic scale (1 = dislike extremely, 9 = like extremely). Low-mineral water was made available for palate cleansing between successive samples.

### 2.10. Data Analysis

All experiments were conducted in triplicate, and results are presented as mean ± standard deviation. Data were analyzed by one-way ANOVA followed by Duncan’s multiple range test at *p* < 0.05 using SPSS (version 23.0) and OriginPro 2022 software.

## 3. Results

### 3.1. Acidification Behavior of Plant-Based Yogurt

**Figure 2:**
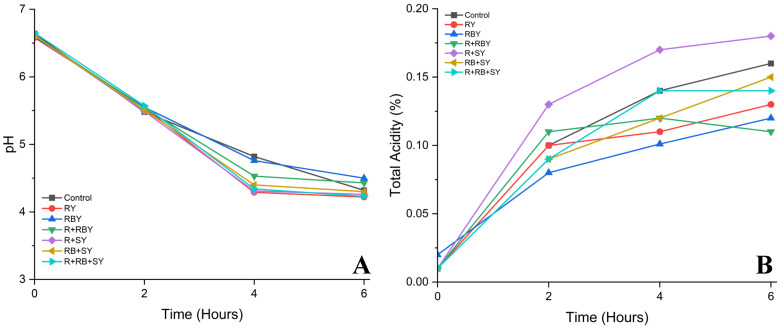
Assessing the pH and percentage of acidity (Lactic acid) of plant-based yogurt. (**A**) pH of plant-based yogurt, (**B**) Total Acidity (%) of plant-based yogurt. Control (soy yogurt), GR (Green rice yogurt), GB (Green rice bran yogurt), GR + GB (Green rice + Green rice bran yogurt), GR + S (Green rice + Soy milk yogurt), GB + S (Rice bran + Soy milk yogurt), GR + GB + S (Green rice + Green rice bran + Soy milk yogurt).

The decrease in pH and the increase in measurable acidity observed during fermentation are primarily due to the metabolic activity of lactic acid bacteria, which convert the available carbohydrates into organic acids, particularly lactic acid [26]. In this study, the rate of acid production indicates a moderate fermentation characteristic, which may be influenced by the amount of fermentable substrate in the rice-based matrix. As shown in Figure 2. Compared to dairy and yogurt systems made from certain legumes, rice-based substrates generally have a lower level of easily fermentable sugars. Therefore, they require enzymatic hydrolysis to release monosaccharides that microorganisms can utilize. The observed trend of acid production in this study is consistent with previous reports on plant-based yogurt, where the gradual decrease in pH and the increase in acidity are related to the metabolic processes of microorganisms during fermentation [11,27,28]. However, when compared to other plant-based systems, such as soy or oat yogurt, the acid production rate in rice-based formulations may be slightly lower, possibly due to differences in carbohydrate composition, protein structure, and nutrient availability. Furthermore, the composition of the substrate plays a crucial role in fermentation efficiency and acid development, as reported by Méndez-Galarraga et al. (2025) [29]. The use of enzyme-assisted extraction in this research is likely to enhance the release of fermentable sugars and bioactive compounds, which support microbial growth and acid production. These research findings underscore the importance of modifying substrates to improve the fermentation efficiency of rice-based alternative yogurt.

### 3.2. Measurement of Physicochemical Properties of Plant-Based Yogurt Products

The type of protein derived from rice was the primary determinant of the physicochemical properties of the plant-based yogurt formulations, indicating the impact of protein source on product quality and structural features [30]. As summarized in Table 2, color parameters (L*, a*, and b*) were notably affected by the inclusion of rice protein extract (GRE) and rice bran protein extract (GBE). Both GR and GB yogurts exhibited significantly lower lightness (L*) values than the soy yogurt control, indicating a darker appearance associated with rice-derived materials. Among all samples, the GB formulation showed the lowest L* value (51.39), which can be attributed to the natural pigments retained in rice bran.

Water-holding capacity (WHC) differed substantially between GR and GB formulations, providing insight into their respective gel network structures. The GR yogurt exhibited a moderate WHC (1.37 g/g), whereas the GB sample showed the lowest WHC (0.91 g/g) among all formulations. The reduced water retention observed in GB is likely related to the presence of rice bran constituents, particularly insoluble dietary fiber, which may disrupt protein–protein interactions and hinder effective water binding within the gel matrix [31].

The high syneresis values observed in GR and GB yogurts (76.22% and 78.30%, respectively) are considerably higher than the typical range reported for acceptable set-type yogurt products (approximately 15–45%), indicating a major limitation in product stability. This behavior can be attributed to the weaker gel network formed by rice proteins, which are predominantly composed of glutelin fractions with limited ability to establish strong protein–protein interactions during acid-induced gelation. In contrast to casein or soy proteins, rice proteins exhibit lower structural cohesion, resulting in poor water-holding capacity. In addition, enzymatic hydrolysis applied during protein extraction may further reduce molecular size and impair network formation [32]. This indicates that these protein networks have limited capacity to retain serum under the tested conditions, similar to the separation of ready-to-drink yogurt [33]. In the case of GB, the particularly high syneresis further supports the notion that bran components, especially insoluble dietary fiber, disrupt continuous gel network formation. Insoluble fibers can sterically hinder protein–protein interactions and introduce heterogeneous, non–gelling domains within the matrix, resulting in a more open structure that cannot effectively trap water [34]. These findings indicate that rice protein and rice bran protein give plant-based yogurt systems distinct physical and chemical characteristics. While both GR and GB formulations exhibited darker color and higher water separation compared to the control group, GB was more affected in terms of reduced lightness, lower water retention capacity, and increased whey separation. These results underscore the importance of protein source selection when developing rice alternative yogurt products and highlight the structural challenges associated with incorporating rice bran protein into plant-based fermentation systems.

### 3.3. Determination of Dry Matter, Ash Content, and Protein in Yogurt

The compositional profile of plant-based yogurt is largely influenced by the types and proportions of ingredients used [35]. In this study, noticeable variations in total solids, ash, and protein contents were observed among the different formulations, reflecting the impact of their distinct raw material compositions. As summarized in Table 3.

Dry matter content ranged from 4.70 to 16.47%, with rice-based formulations showing the greatest variation. GB yogurt exhibited the lowest value (4.70%), which may be attributed to the higher fiber content and lower solid recovery associated with rice bran. GR yogurt showed a slightly higher dry matter content (5.66%), while the combined GR + GB formulation reached 7.52%. All rice-based samples remained lower than the control (13.94%), likely reflecting differences in compositional characteristics and extractable solids compared with the soy-based system. These findings indicate that the nature of the raw material, degree of processing, and structural complexity of rice-derived ingredients play important roles in determining total solids content within the fermented yogurt matrix.

Ash content showed only minor variation among formulations. The control sample had the highest value (0.13%), while the remaining samples ranged from 0.05 to 0.08% with no statistically significant differences. Although rice bran is generally recognized as a good source of minerals, mineral retention in the final yogurt matrix may have been affected by processing and fermentation conditions. Overall, the limited variation in ash content indicates that differences in raw materials had only a modest impact on the mineral composition of the finished products [35].

Protein content varied clearly among formulations, consistent with differences in raw material composition. Among the rice-based yogurts, GB showed the highest protein content (24.22%), followed by GR + GB (22.40%) and GR (20.92%). All three samples exhibited protein contents approximately 1.93–2.23-fold higher than the control (10.85% DW), confirming that both green rice and rice bran extracts significantly enhanced protein levels in the yogurt system, despite their lower dry matter content, highlighting the potential of rice bran protein as a functional protein source [36]. These results demonstrate that green rice and rice bran extracts distinctly influence yogurt composition. GB contributed the strongest protein enrichment, GR provided moderate protein with slightly higher solids than GB, and the GR + GB system offered a complementary balance of both components. This highlights the potential to tailor nutritional composition in rice-based fermented products through strategic selection and combination of rice-derived ingredients.

### 3.4. Measures the Number of Viable Cells in Plant-Based Yogurt Products

Variations in viable cell counts and texture among the plant-based yogurts can be attributed to differences in protein structure compared with dairy systems. Milk casein readily forms a compact gel network during acidification, producing the typical curd structure of conventional yogurt [37]. As summarized in Table 3, in contrast, soy and rice proteins differ in amino acid composition and molecular conformation, leading to alternative gelation behavior and softer textures [36]. Microbiological analysis showed slightly higher viable counts in rice- and rice bran–based formulations compared with the control. The control sample reached 9.90 log CFU/mL, whereas rice-containing yogurts ranged from 9.94 to 10.07 log CFU/mL. The improved survival may be associated with the potential production of exopolysaccharides (EPS) by certain lactic acid bacteria, as reported in previous studies, which can contribute to improved viscosity, matrix stability, and microbial protection [38]. The viable cell counts observed in this study (approximately 9.9–10.1 log CFU/mL) are relatively high compared to commonly reported values for plant-based yogurt systems, which typically range from 7 to 9 log CFU/mL. This indicates robust microbial growth and effective fermentation under the conditions applied [39].

Such high cell densities may contribute positively to product stability by promoting rapid acidification and inhibiting the growth of undesirable microorganisms. In addition, viable counts above 6–7 log CFU/mL are generally considered sufficient to confer potential probiotic benefits, suggesting that the developed products may have functional value [40]. It also promotes adhesion to the food matrix and intestinal surfaces [41] and improves resistance to environmental stresses such as heat, drying, and oxidation [42] these effects support LAB viability and contribute to the stability and probiotic potential of plant-based yogurt systems. Lactic acid bacteria primarily utilize fermentable carbohydrates as their main energy source during fermentation. Although the specific sugar composition of the rice- and rice bran–based substrates was not characterized in this study, it is well established that variations in carbohydrate profiles can influence microbial growth, acidification, and overall fermentation performance. This relationship may contribute to the differences observed in pH reduction and titratable acidity among the formulations. Similar observations have been reported in previous studies on plant-based yogurt systems, where substrate composition plays a key role in fermentation behavior [43,44,45]. However, this study has certain limitations, particularly the absence of molecular identification techniques, such as 16S rRNA gene sequencing, for precise microbial characterization. Therefore, future studies incorporating these methods are warranted to obtain more comprehensive and reliable results.

### 3.5. Texture of Plant-Based Yogurts

Texture is a critical quality attribute of yogurt, reflecting the formation and integrity of the protein gel network and directly influencing consumer acceptance [46]. As shown in Table 3, significant differences in textural parameters were observed among formulations (*p* < 0.05). The soy-based control exhibited the highest firmness (5.41 g) and consistency (13.29 g), indicating the formation of a dense and well-connected gel network under the applied fermentation conditions [38]. In contrast, GR and GB formulations, including their blended variants, showed lower firmness (0.20–0.60 g) and consistency (0.59–1.84 g), suggesting the development of softer and less rigid gel structures. These differences can be explained by the intrinsic structural and functional properties of the proteins. Soy proteins are predominantly globular storage proteins, such as glycinin and β-conglycinin, which readily undergo unfolding and aggregation near their isoelectric point during acidification, facilitating the formation of a continuous and elastic three-dimensional network [47]. In contrast, rice proteins are mainly composed of glutelin, which exhibits lower solubility and a more limited ability to unfold and reorganize into cohesive networks under acidic conditions [48]. As a result, rice protein systems tend to form weaker and more particulate gel structures, leading to reduced firmness and consistency. In addition, the enzymatic extraction process likely induced partial hydrolysis of rice proteins, resulting in reduced molecular size and altered protein conformation. This can limit protein–protein interactions and network formation during acidification, further contributing to the softer gel characteristics observed in GR and GB formulations [49,50]. The GB formulation demonstrated a distinct textural profile, which may be attributed to the presence of insoluble dietary fiber and associated bioactive compounds in rice bran. These components can influence matrix organization by enhancing water immobilization and increasing resistance to flow, as reflected by the relatively higher consistency observed in GB samples [11,51]. Furthermore, the potential contribution of exopolysaccharides (EPS) produced by lactic acid bacteria during fermentation may also play a role in modulating texture. EPS are known to enhance viscosity, improve water retention, and stabilize the gel matrix, thereby influencing mouthfeel and overall sensory perception [52]. Overall, the observed textural variations highlight the influence of protein composition, structural characteristics, and processing conditions on gel formation behavior in plant-based yogurt systems.

### 3.6. Rheological of Plant-Based Yogurts

**Figure 3:**
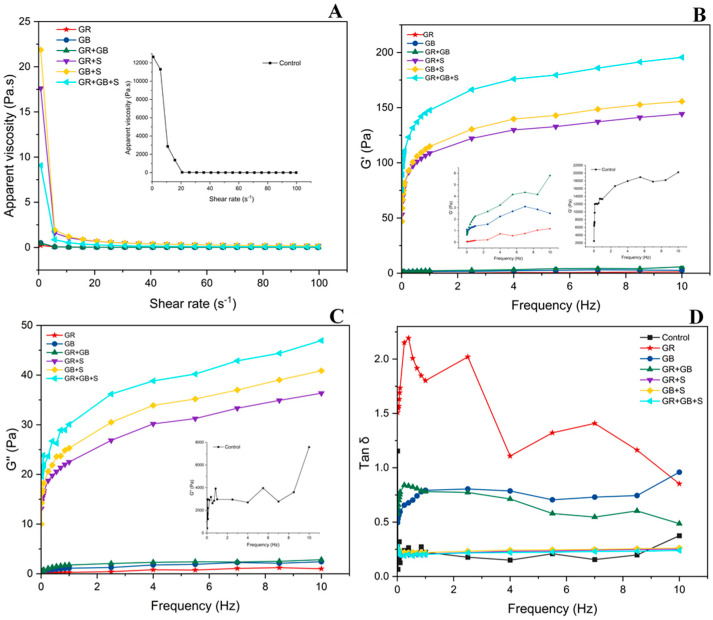
Rheological of plant-based yogurts. (**A**) Apparent viscosity as a function of shear rate. (**B**) Storage modulus (G′) as a function of frequency. (**C**) Loss modulus (G″) as a function of frequency. (**D**) Tan δ as a function of frequency. Values represent mean ± SD (*n* = 3). Control (soy yogurt), GR (Green rice yogurt), GB (Green rice bran yogurt), GR + GB (Green rice + Green rice bran yogurt), GR + S (Green rice + Soy milk yogurt), GB + S (Rice bran + Soy milk yogurt), GR + GB + S (Green rice + Green rice bran + Soy milk yogurt).

Rheological measurements provide insight into the structural organization of yogurt by describing the strength and deformation behavior of the gel network under applied shear [1]. As shown in Figure 3, all formulations exhibited typical viscoelastic characteristics, with storage modulus (G′) consistently higher than loss modulus (G″) across the tested frequency range, indicating predominantly elastic, gel-like structures. Tan δ values further described the balance between elastic and viscous contributions [53,54,55].

All samples demonstrated shear-thinning behavior, with apparent viscosity decreasing as shear rate increased. Rice-based formulations (GR and GB) displayed lower apparent viscosity and reduced G′ and G″ values compared with the control, reflecting differences in network rigidity rather than structural discontinuity. Despite these differences in magnitude, G′ remained higher than G″ throughout the frequency sweep, confirming the formation of coherent viscoelastic networks. Based on storage modulus values across the frequency range, gel strength followed the order: Control > GR + GB + S > GR + S > GB + S > GR + GB > GB > GR. The GR + GB formulation showed improved viscoelastic balance relative to single-component rice systems, suggesting complementary interactions between rice grain and rice bran constituents that contributed to enhanced structural organization within the fermented matrix. This behavior is consistent with protein aggregation and matrix restructuring during acidification, as reported in previous studies [56]. Tan δ values (Figure 3D) remained below 1 for all formulations, confirming elastic-dominated responses characteristic of structured yogurt gels. Rice-based samples exhibited slightly higher tan δ values than soy-containing systems, indicating a relatively greater viscous contribution; however, all formulations maintained stable gel-like behavior across the tested frequency range [57,58]. Overall, the results demonstrate that rice-derived proteins and rice bran components are capable of forming stable viscoelastic networks in fermented yogurt systems. While the magnitude of rheological parameters varied among formulations, all rice-based systems exhibited coherent gel structures, supporting their applicability as functional ingredients in plant-based fermented products.

### 3.7. Bioactive Compounds and Antioxidant Activity of Plant-Based Yogurt

Many types of yogurt have added natural extracts to increase the bioactive compounds in the product, including those from rice grains and rice bran [59,60]. As summarized in Table 4, pronounced differences in bioactive compound content and antioxidant activity were observed among the plant-based yogurt formulations, underscoring the distinct contributions of rice-derived ingredients.

Total phenolic content (TPC) ranged from 19.66 to 29.75 mg GAE/100 g DW. GR exhibited the highest TPC (29.75 mg GAE/100 g DW), followed by GB (28.87 mg GAE/100 g DW) and GR + GB (27.84 mg GAE/100 g DW), all significantly higher than the control. This confirms that both green rice and rice bran extracts effectively enriched phenolic compounds in the yogurt matrix [61]. A more pronounced effect was observed for total flavonoid content (TFC). The GR + GB formulation showed the highest TFC (883.40 mg QE/100 g DW), exceeding both GR (536.78 mg QE/100 g DW) and GB (740.20 mg QE/100 g DW), indicating a complementary interaction between rice grain and bran fractions that enhanced flavonoid accumulation. This enhancement is likely attributable to matrix complementation between rice grain and bran components. In this system, flavonoids associated with bran fiber structures and protein bound phenolics originating from the grain may be simultaneously released during enzymatic extraction and fermentation [10,62].

Antioxidant activity followed a similar trend. GR exhibited the strongest DPPH radical-scavenging activity (199.58 mg AA/100 g DW) and FRAP value (1403.68 mg FeSO_4_/100 g DW), while GB also demonstrated high antioxidant capacity. The GR + GB formulation maintained substantial antioxidant activity (DPPH: 190.17 mg AA/100 g DW; FRAP: 1259.55 mg FeSO_4_/100 g DW), confirming that the combined rice grain–bran system provides a broad spectrum of bioactive compounds. These findings align with previous studies reporting enhanced antioxidant activity in yogurt systems enriched with rice, rice bran, or anthocyanin-rich rice extracts [63]. Rice bran fortified yogurt demonstrated higher total phenolic content and antioxidant capacity, consistent with the findings of Ariyani et al. [64], who reported increased levels of bioactive compounds following enrichment. Similar observations were described by Siripan et al. [10], where enzymatic extraction significantly enhanced the release of active compounds. These findings indicate that incorporating both green rice protein and rice bran can effectively improve the bioactive composition and antioxidant potential of plant-based yogurt formulations, highlighting their value as functional ingredients in fermented products.

### 3.8. Descriptive Sensory of Plant-Based Yogurt Products

**Figure 4:**
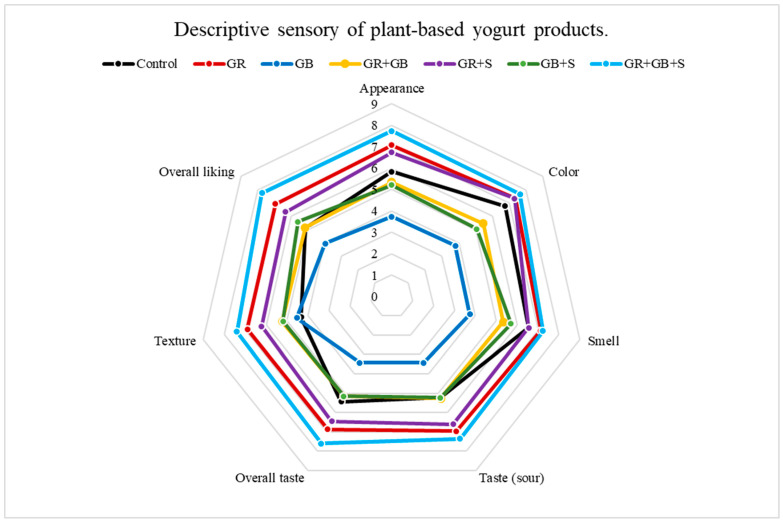
Descriptive sensory of plant-based yogurt products. A descriptive sensory evaluation of yogurt samples was conducted by 30 untrained assessors (aged 18–45 years). Values represent mean ± SD (n = 30). Control (soy yogurt), GR (Green rice yogurt), GB (Green rice bran yogurt), GR + GB (Green rice + Green rice bran yogurt), GR + S (Green rice + Soy milk yogurt), GB + S (Rice bran + Soy milk yogurt), GR + GB + S (Green rice + Green rice bran + Soy milk yogurt).

Sensory evaluation plays an essential role in determining consumer acceptance, as it captures perceptions of appearance, color, aroma, taste, and texture that cannot be fully explained by instrumental measurements alone [65]. As shown in Figure 4, significant differences were found between the formulations, reflecting the impact of rice-derived proteins on overall product perception.

Yogurts formulated with green rice protein extract (GRE) and green rice bran extract (GBE) displayed sensory profiles that differed significantly from the soy-based control. The GR yogurts sample received high ratings for appearance, color, taste, and texture, reflecting a mild flavor and smooth mouthfeel associated with rice protein incorporation, and achieved the high preference score. Although GB yogurts had a darker color due to natural bran pigments, this visual difference did not substantially reduce overall acceptance. Aroma scores were comparable among all formulations, indicating that the rice and bran ingredients did not contribute undesirable odors. In addition, GR yogurts were perceived as having a more favorable texture than the control, consistent with its measured gel strength and water-holding capacity. Overall preference ratings indicated that rice-based yogurts, particularly GR yogurts, were accepted at levels comparable to or exceeding those of control. These results highlight the potential of green rice protein and rice bran as effective primary ingredients for developing sensory-acceptable fermented yogurt alternatives derived from rice [66].

## 4. Conclusions

This study demonstrates that proteins derived from green rice grains and rice bran, obtained through enzyme-assisted extraction, effectively contribute to the functional, nutritional, and bioactive characteristics of rice-based plant yogurt systems. Rice-derived proteins imparted distinctive physicochemical and rheological properties, together with measurable antioxidant activity and acceptable sensory attributes, reflecting their inherent compositional and structural features. Green rice grain and green rice bran proteins successfully formed coherent viscoelastic gel networks under acidic fermentation conditions, exhibiting characteristic texture and flow behavior typical of structured yogurt systems. Differences in modulus magnitude and viscosity among formulations indicate tunable structural properties, allowing flexibility in product design depending on desired texture and consistency. Blended formulations further demonstrated that combining rice components can enhance structural balance and overall acceptability while maintaining the intrinsic nutritional and bioactive identity of the rice-based matrix. These findings highlight the technological feasibility of utilizing rice-derived proteins as primary functional ingredients in fermented plant-based products.

Overall, this work advances the understanding of rice proteins in acid-induced gel systems and supports their application in the development of diversified, rice-based plant yogurts with functional, nutritional, and market relevance.

## Figures and Tables

**Table 1:** Plant-based yogurt production process.

Formula	Component (%)			
	Soy Milk	Green Rice Extract (GE)	Green Rice Bran Extract (GBE)	Other Ingredients
Control	92.90	0.00	0.00	Sugar 6.00 Guar gum 0.10 Starter culture 1.00
GR	0.00	92.90	0.00	
GB	0.00	0.00	92.90	
GR + GB	0.00	46.45	46.45	
GR + S	46.45	46.45	0.00	
GB + S	46.45	0.00	46.45	
GR + GB + S	46.45	23.23	23.23	

Formulation of plant-based yogurt analogues prepared from soy milk (S), green rice extract (GE), and green rice bran extract (GBE). The total base composition was fixed at 92.90% in all formulations, while the remaining components (sugar, guar gum, and starter culture) were kept constant. Binary and ternary blends were formulated using equal or proportionally distributed ratios to enable systematic comparison of the functional properties and interactions among different protein sources. Control (soy yogurt), GR (Green rice yogurt), GB (Green rice bran—yogurt), GR + GB (Green rice + Green rice bran yogurt), GR + S (Green rice + Soy milk yogurt), GB + S (Rice bran + Soy milk yogurt), GR + GB + S (Green rice + Green rice bran + Soy milk yogurt).

**Table 2:** Physicochemical properties of plant-based yogurt products.

Sample	Side View	Color			WHC (g/g)	Syneresis (%)
		L*	a*	b*		
Control	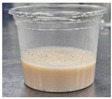	77.27 ± 0.62 ^a^	−3.08 ± 0.15 ^e^	11.57 ± 1.09 ^a^	1.92 ± 0.58 ^ab^	39.06 ± 0.75 ^c^
GR	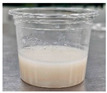	61.57 ± 0.48 ^e^	−1.57 ± 0.07 ^c^	2.13 ± 0.23 ^c^	1.37 ± 0.35 ^bc^	76.22 ± 1.64 ^a^
GB	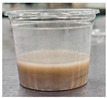	51.39 ± 0.68 ^g^	0.57 ± 0.08 ^a^	11.52 ± 0.50 ^a^	0.91 ± 0.08 ^c^	78.30 ± 2.58 ^a^
GR + GB	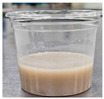	55.57 ± 0.55 ^f^	−0.46 ± 0.33 ^b^	7.79 ± 0.32 ^b^	1.23 ± 0.62 ^bc^	78.26 ± 3.59 ^a^
GR + S	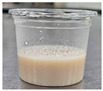	70.01 ± 0.50 ^b^	−2.93 ± 0.13 ^e^	3.82 ± 3.08 ^c^	2.48 ± 0.6 ^a^	44.65 ± 3.27 ^b^
GB + S	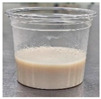	66.52 ± 0.04 ^d^	−1.77 ± 0.04 ^cd^	9.82 ± 0.07 ^ab^	2.5 ± 0.69 ^a^	44.38 ± 2.25 ^b^
GR + GB + S	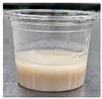	67.48 ± 0.06 ^c^	−1.96 ± 0.01 ^d^	8.03 ± 0.03 ^b^	1.30 ± 0.26 ^bc^	47.82 ± 5.00 ^b^

Values are shown as mean ± SD (*n* = 3), with significant differences among treatments within each column indicated by distinct letters (^a, b, c…^) at the 0.05 significance level. Control (soy yogurt), GR (Green rice yogurt), GB (Green rice bran yogurt), GR + GB (Green rice + Green rice bran yogurt), GR + S (Green rice + Soy milk yogurt), GB + S (Rice bran + Soy milk yogurt), GR + GB + S (Green rice + Green rice bran + Soy milk yogurt).

**Table 3:** Chemical Composition, Textural, and Visual Characteristics of Plant-Based Yogurts.

Sample	Chemical Composition			Log CFU/mL of Sample.	Texture Analysis		
	Dry Matter (%)	Ash (% DW)	Protein (% DW)	Solution 10^−8^	Cohesiveness (g)	Firmness (g)	Consistency (gs)
Control	13.94 ± 1.60 ^b^	0.13 ± 0.02 ^a^	10.85 ± 0.96 ^e^	9.90 ± 0.01 ^c^	−0.54 ± 0.27 ^c^	5.41 ± 1.97 ^a^	13.29 ± 21.23 ^a^
GR	5.66 ± 0.39 ^e^	0.08 ± 0.01 ^bc^	20.92 ± 1.06 ^d^	10.01 ± 0.02 ^b^	−0.01 ± 0.01 ^a^	0.24 ± 0.01 ^b^	1.31 ± 0.38 ^b^
GB	4.70 ± 0.09 ^e^	0.05 ± 0.01 ^c^	24.22 ± 2.13 ^bc^	9.86 ± 0.01 ^d^	−0.01 ± 0.01 ^a^	0.24 ± 0.01 ^b^	1.84 ± 0.77 ^b^
GR + GB	7.52 ± 0.23 ^d^	0.07 ± 0.02 ^bc^	22.40 ± 0.56 ^cd^	9.94 ± 0.02 ^c^	−0.01 ± 0.00 ^a^	0.20 ± 0.01 ^b^	1.07 ± 0.04 ^b^
GR + S	16.47 ± 0.11 ^a^	0.08 ± 0.02 ^b^	24.94 ± 1.75 ^ab^	10.07 ± 0.01 ^a^	−0.23 ± 0.01 ^b^	0.46 ± 0.01 ^b^	1.54 ± 1.56 ^b^
GB + S	11.15 ± 0.58 ^c^	0.05 ± 0.01 ^bc^	26.80 ± 0.93 ^a^	10.03 ± 0.02 ^b^	−0.27 ± 0.03 ^b^	0.60 ± 0.03 ^b^	0.59 ± 0.02 ^b^
GR + GB + S	15.42 ± 0.09 ^a^	0.06 ± 0.01 ^bc^	22.65 ± 1.00 ^bcd^	10.03 ± 0.01 ^b^	−0.20 ± 0.01 ^b^	0.26 ± 0.01 ^b^	1.36 ± 0.11 ^b^

Values represent mean ± SD (*n* = 3 in Chemical composition and *n* = 5 in Texture Analysis), with significant differences among treatments within each column indicated by distinct letters (^a, b, c…^) at the 0.05 significance level. Control (soy yogurt), GR (Green rice yogurt), GB (Green rice bran yogurt), GR + GB (Green rice + Green rice bran yogurt), GR + S (Green rice + Soy milk yogurt), GB + S (Rice bran + Soy milk yogurt), GR + GB + S (Green rice + Green rice bran + Soy milk yogurt).

**Table 4:** Characterization of bioactive compounds and antioxidant activity in plant-based yogurt.

Sample	TPC (mg GAE/100 g DW)	TFC (mg QE/100 g DW)	DPPH (mg AA/100 g DW)	FRAP (mg FeSO_4_/100 g DW)
Control	19.66 ± 0.38 ^f^	203.40 ± 3.93 ^f^	172.64 ± 0.15 ^g^	708.24 ± 3.01 ^g^
GR	29.75 ± 0.06 ^a^	536.78 ± 4.36 ^d^	199.58 ± 0.20 ^a^	1403.68 ± 3.33 ^a^
GB	28.87 ± 0.11 ^b^	740.20 ± 3.06 ^b^	195.56 ± 0.41 ^b^	1326.80 ± 3.86 ^c^
GR + GB	27.84 ± 0.07 ^c^	883.40 ± 1.90 ^a^	190.17 ± 0.15 ^d^	1259.55 ± 6.89 ^d^
GR + S	22.69 ± 0.15 ^d^	427.94 ± 2.93 ^e^	191.44 ± 0.42 ^c^	1218.08 ± 2.94 ^e^
GB + S	22.55 ± 0.07 ^d^	433.14 ± 2.86 ^e^	187.24 ± 0.15 ^e^	1037.25 ± 5.03 ^f^
GR + GB + S	21.05 ± 0.05 ^e^	579.52 ± 4.79 ^c^	185.49 ± 0.24 ^f^	1383.51 ± 2.22 ^b^

Values are shown as mean ± SD (*n* = 3), with significant differences among treatments within each column indicated by distinct letters (^a, b, c…^) at the 0.05 significance level. Control (soy yogurt), GR (Green rice yogurt), GB (Green rice bran yogurt), GR + GB (Green rice + Green rice bran yogurt), GR + S (Green rice + Soy milk yogurt), GB + S (Rice bran + Soy milk yogurt), GR + GB + S (Green rice + Green rice bran + Soy milk yogurt).

## Data Availability

The original contributions presented in this study are included in this article. Further inquiries can be directed to the corresponding author.
